# Is Thermosensing Property of RNA Thermometers Unique?

**DOI:** 10.1371/journal.pone.0011308

**Published:** 2010-07-02

**Authors:** Premal Shah, Michael A. Gilchrist

**Affiliations:** 1 Department of Ecology and Evolutionary Biology, University of Tennessee, Knoxville, Tennessee, United States of America; 2 National Institute for Mathematical and Biological Synthesis, Knoxville, Tennessee, United States of America; Institute of Protein Research, Russian Academy of Sciences, Russian Federation

## Abstract

A large number of studies have been dedicated to identify the structural and sequence based features of RNA thermometers, mRNAs that regulate their translation initiation rate with temperature. It has been shown that the melting of the ribosome-binding site (RBS) plays a prominent role in this thermosensing process. However, little is known as to how widespread this melting phenomenon is as earlier studies on the subject have worked with a small sample of known RNA thermometers. We have developed a novel method of studying the melting of RNAs with temperature by computationally sampling the distribution of the RNA structures at various temperatures using the RNA folding software Vienna. In this study, we compared the thermosensing property of 100 randomly selected mRNAs and three well known thermometers - *rpoH*, *ibpA* and *agsA* sequences from *E. coli*. We also compared the *rpoH* sequences from 81 mesophilic proteobacteria. Although both *rpoH* and *ibpA* show a higher rate of melting at their RBS compared with the mean of non-thermometers, contrary to our expectations these higher rates are not significant. Surprisingly, we also do not find any significant differences between *rpoH* thermometers from other 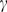-proteobacteria and *E. coli* non-thermometers.

## Introduction

Many microorganisms live in a variable environment. They have evolved a variety of mechanisms to sense changes in their environment and alter their gene expression in response to these changes. Regulatory proteins often play a role in controlling the level of transcription and translation of other genes. However, in certain cases post-transcriptional mechanisms, such as changes in mRNA conformation, are known to influence gene expression. In some prokaryotes, reaction to changes in the temperature is thought to be mediated by one such class of mRNAs called RNA thermometers [1]–[5]. At lower temperatures, the thermosensing region in these sequences adopts a secondary structure that sequesters the ribosome binding site (RBS) of a gene, hence interfering with translation initiation by the ribosome. At higher temperatures, this thermosensing region upstream of the coding sequence melts, increasing the accessibility of the RBS leading to an increase in the initiation of translation and, in turn, its protein production rate [1], [4]–[6].

Previous work on RNA thermometers has focused primarily on understanding and identifying their sequence based features and residues important for thermosensing [1]–[3], [6]. Time elapsed spectral studies [7] and mutational analyses [1]–[3] of the thermometer genes have been used to identify regions, which play a crucial part in the thermosensing property. For instance, in one of the most studied RNA thermometer called the ROSE (Repression Of heat-Shock gene Expression) element, a guanine residue at position 83, paired opposite the Shine-Dalgarno (SD) sequence in a hairpin structure is known to play a prominent role in the ability of the mRNA to change its expression with temperature [4].

Although these studies provide insights into the mechanisms by which specific thermometers function, little is known as to how widespread these mechanisms are. The fraction of genes in a genome that possess an ability to regulate their translation by thermosensing or a similar mechanism is unknown. More importantly, because the above studies do not include non-thermometers as controls, it is difficult to ascertain if RNA thermometers are a special class of molecules different from other RNAs. Since it is not feasible to perform mutational or spectral studies on every gene to identify whether it behaves as an RNA thermometer, computational tools need to be developed to provide these insights. We here propose a computational approach to characterize RNA thermometers and ask how they differ from non-thermometers in their ability to melt with increasing temperature. Understanding the melting potential of non-thermometers should aid in understanding the adaptive features of RNA thermometer sequences. We focus specifically on the ability of genes to change their expression by modifying the accessibility of RBS, or in other words, ‘RBS exposure’.

**Figure 1:**
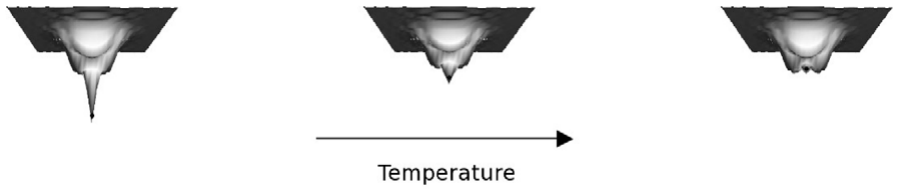
Effect of temperature on the energy landscape. As temperature increases, the probability of finding an mRNA in its most stable state decreases. This is because at higher temperatures, molecules have more energy enabling them to spend more time in higher energy states. Also, at higher temperatures, as the energy landscape becomes flatter, uniqueness of the stable state may also be lost [11].

Earlier attempts to identify potential RNA thermometers have focused on search patterns based on similarities in the secondary structure of the mRNAs [8], [9]. However, the use of a fixed length sequence for secondary structure limits the utility of this approach. For instance, sequences that differ by only a single nucleotide in their lengths can have drastic differences in their predicted secondary structures [10]. Secondly, most studies when looking at secondary structures of RNAs use mainly the least free energy (LFE) structures. Although, this approach of using the most stable structures has proved useful, there are certain shortcomings when used for characterizing RNA thermometers. It has been shown that as temperature increases, the overall probability and uniqueness of finding a structure in its LFE state decreases [11], [12]. Thus, such an approach could lead to spurious results as the energy landscape of the molecule evolves with temperature (Fig. 1). In addition, looking at LFE structures at a single temperature alone provides no means of quantifying the effect of temperature on the structure. Finally, any pattern-based approach to finding thermometers is restrictive, as it does not take into account novel structures that might be thermosensing.

Here we propose a novel method of quantitatively studying secondary structures of RNAs that addresses all of the above shortcomings. This method explores the ability of mRNAs to change their rate of translation initiation with temperature. We see this approach as complementary to experimental studies in the field of RNA structures.

## Methods

We used the RNAsubopt package from RNA folding software Vienna [13] to predict secondary structures of the RNAs. This package was used to sample 1000 secondary structures at each temperature for every gene from the entire distribution of structures at that temperature. The sampling of sub-optimal structures is important because RNA secondary structures with very similar free energies can have drastic differences in their secondary structures [12], which might not be captured when looking at the structure with least energy in isolation. The program RNAsubopt generates structures with *probabilities equal to their Boltzmann weights* via stochastic backtracking in the partition function [14]. Since these structures are drawn based on their Boltzmann weights, the entire ensemble of 1000 structures can be viewed as a time ensemble, i.e., the probability of finding a particular structure in our ensemble is proportional to the amount of time the RNA is found to be in that structure. Thus, stable structures would have higher Boltzmann weights and the RNA would spend a greater amount of time in that structure.

In order to understand the effect of temperature on gene expression as measured by RBS exposure, we randomly selected 100 non-thermometer mRNAs from the *E. coli* genome (see Supporting Table S1) as well as *rpoH* mRNA sequence, a known thermometer, from 81 mesophilic 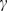-proteobacteria for this study (see Supporting Table S2). Transcript start and end positions for *E. coli* genes were obtained from the RegulonDB database [15]. Information regarding the position of RBS on the transcript was obtained from the *flexrbs* dataset [16] (see Supporting Table S1). We used the entire length of the mRNA (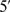 UTR+ORF+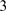 UTR) to generate the sub-optimal structures. This was done for the following reasons. The secondary structure of mRNA is highly dependent on the length of the sequence used for simulation [10]. Using a shorter length may prevent detection of any long-range interactions that might be crucial for the stability, and function of the RNA molecule. Moreover, although translation is coupled with transcription in prokaryotes, the half-life of an mRNA is considerably longer than the time required for translation [17]–[19] and hence the mRNA transcript would spend most of its time as a full-length sequence. Thus, we argue that the secondary structure of the mRNA is better simulated by using the entire mRNA length for our purposes. We also check whether our results are robust to using an mRNA sequence of length 150 nucleotides centered around the RBS (see Supporting Figure S1). Of the 100 genes from *E. coli*, 56 genes were part of operons. In the case of operons, we simulated the entire mRNA sequence but categorized multiple RBSs within an operon individually.

We simulated 1000 secondary structures of each mRNA at 7 different temperatures ranging from 25

C to 50

C. All other parameters in RNAsubopt were used at default values. In order to quantify the openness of RNA, we used a sliding window length of 7 bases to estimate the fraction of simulated structures in which none of the bases in that window were involved in base pairing. A window length of 7 was chosen because the Shine-Dalgarno sequence/RBS in *E. coli* varies from 4–7 bases [16], [20]. Changing the window length from 5 bases to 10 bases still resulted in the same qualitative behavior. However, as one would expect, because of the categorical nature of the data (open or close), the fraction of open or melted windows in the structure decreased with window length.

An alternative to sampling structures based on Boltzmann's distribution is to estimate the least free energy (LFE) structures by constraining the RBS in the open conformation [21]. The LFE of the constrained and the unconstrained structures can then be used to estimate probability of openness of the RBS. However, as mentioned earlier, with and increase in temperature, the overall probability and uniqueness of finding a structure in its LFE state decreases [11], [12]. Thus, such a method severely limits the ability to compare the probability of openness across temperatures.

In order to compare the probability of openness across temperatures, we fitted a logistic model to the fraction of open windows as a function of temperature.
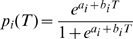
(1)where 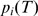 is the probability of finding the window at position 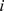 in a gene, open at temperature 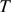 (
C), 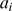 and 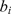 are the intercept and slope parameters of how the log-odds of finding an open window at position 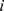, 
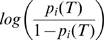
, changes with temperature. The ratio 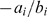 indicates the temperature at which the probability of openness of a window is 0.5. Although the probability of openness of RBS is positively correlated with protein expression, the exact relationship between the two is unknown.

**Figure 2:**
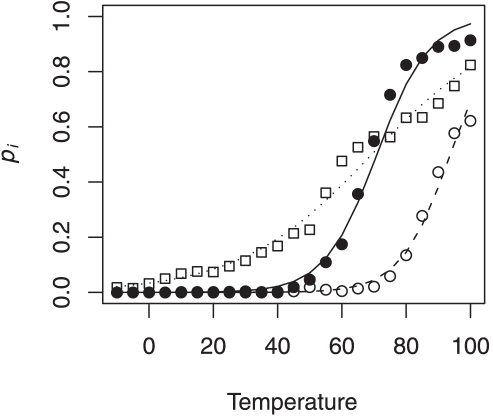
Fitting logistic regression. The solid circles indicate the probability of openness, 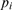 at the RBS of *rpoH* gene. The open circles and squares represent two randomly chosen windows within *rpoH*. The best fit lines of the logistic regression are given by the solid line for RBS and dashed and dotted line for the randomly chosen windows.

We find that the logistic model serves as a reasonable descriptor of RNA melting (Fig. 2). At very low temperatures, we expect most of the bases in the RNA to be paired with other bases. Hence, the probability of openness of a window would approach 0. At very high temperatures, the free energy of base-pairing decreases and most bases would be unpaired causing the probability of openness to approach 1. Thus in a specific range of temperatures, determined by the parameters 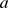 and 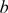, we can potentially see a transition between the two states. However, we restrict our simulations to the biological relevant temperature range for mesophiles (25

C–55

C). In this study, we are primarily interested in the parameter 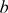, which describes the rate of change of openness with temperature. For each window within each gene, the Maximum-Likelihood Estimates (MLE) of 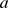 and 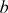 were calculated using R [22].

## Results

### Capturing the behavior of RNA thermometers

**Figure 3:**
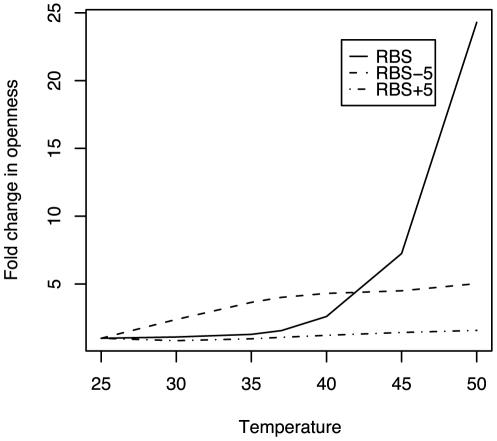
Fold-change in the openness of the RBS and regions 5 bases upstream and downstream of it with temperature. The fold change is with respect to the openness at 25

To show that our method is capable of capturing the increase in openness of the RBS of an RNA thermometer, we used the *rpoH* gene sequence of *E. coli*. The *rpoH* gene is a 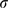-factor involved in the up-regulation of the heat-shock proteins during higher temperatures. It is one of the most studied RNA thermometers [1]–[3]. Fig. 3 illustrates how as temperature increases, the RBS of *rpoH* shows a much higher fold-change in openness as compared to the regions flanking it. The openness of the RBS at 50

C was 25 folds higher than at 25

**Figure 4:**
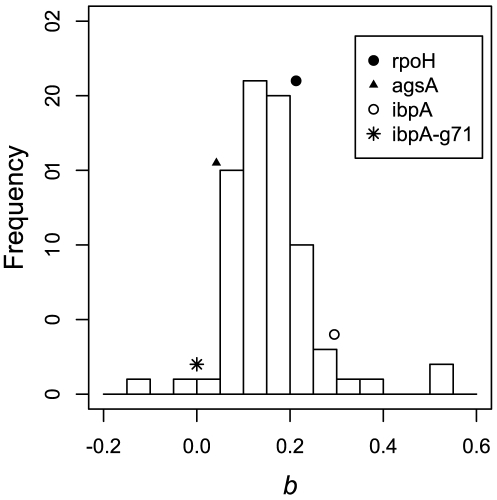
The distribution of MLE estimates of 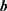 of the 76 genes that differed significantly from zero in *E. coli*. *rpoH*, *ibpA* and *agsA* genes show an increase in openness with temperature with 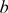 values 0.213, 0.295 and 0.042, respectively. However, none of these values are significantly higher than the mean of the distribution (*Wilcox test*, 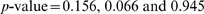, respectively). In addition, when the base G71 is removed from *ibpA* sequence, the MLE estimate of 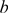 reduces to 0.

C. These results are consistent with the idea that the RBS of a gene might be under stronger selection to increase its openness with temperature. We were also able to replicate the experimental results of [8] where they showed that the deletion of guanine at position 71 (G71) of the gene *ibpA* in *E. coli*, resulted in a loss of thermosensing activity. Fig. 4 shows that both the RNA thermometers *rpoH* and *ibpA* have a higher rate of increase in their RBS exposure compared to the mean of the randomly selected 100 *E. coli* genes. However, the MLE of 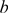 drops to 0 when G71 is removed from the *ibpA* gene sequence, as we did not observe a single open window in 1000 runs at all temperatures between 25

C and 50

C at that position.

C. The RBS of *rpoH* gene has a much higher increase in openness with temperature than the regions around it.

### Comparing thermometers and non-thermometers

**Figure 5:**
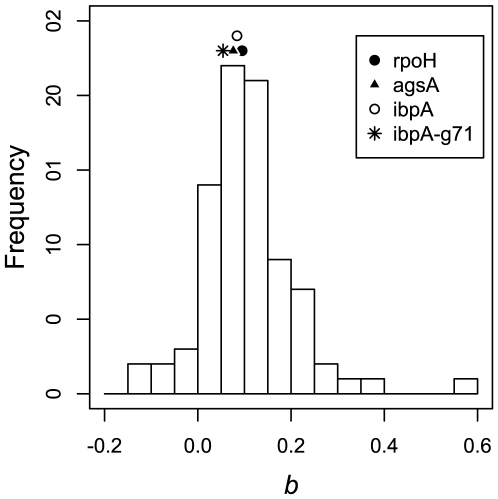
The distribution of MLE estimates of 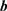 at the start codon (ATG) of the 85 genes that significantly differ from zero in *E. coli*. *rpoH*, *ibpA* and *agsA* genes show an increase in openness with temperature with 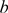 values 0.095, 0.084 and 0.076, respectively. However, all the values are less than mean of the distribution. Also, when the base G71 is removed from the *ibpA* sequence, the MLE estimate of 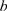 reduces to 0.054. The results are consistent with what is observed at the RBS window.

When the rate of openness of RBS, 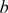 was compared across the 100 genes, we found that 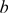 values were not significantly greater than zero for 24 genes at 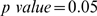. This implies that a small fraction of genes did not show a significant change in openness of its RBS with temperature over the range of temperatures considered. This is surprising because if RNA thermometers were a rare class of mRNAs, then this number would have been far higher. The distribution of the 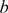 values for the remaining 76 genes is shown in Fig. 4. Since the distribution of 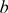 values is not a Gaussian distribution (*Shapiro-Wilk test*, 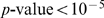), non-parametric tests were employed for further statistical analyses. Although the two of the three RNA thermometers, *rpoH* and *ibpA* had a higher 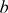 value than the mean of the entire distribution (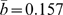), these higher rates of openness were not significant (*Wilcox test*, 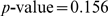 and 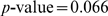, respectively). Interestingly, we find that RNA thermometer *agsA* had a 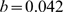, which, although positive, is lower than the mean of the distribution of 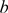 values of non-thermometers. We also show that there is no qualitative difference in our results when considering only 150 nucleotides of the mRNA centered around the RBS (see Supporting Figure S1). This result did not change even after including non-significant values of 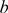 in the above test. This indicates that RNA thermometers do not differ significantly from non-thermometers in increasing the openness of RBS with temperature. It argues that every RNA molecule has an inherent tendency to melt with temperature, albeit to varying degree. These results are also consistent when considering the window spanning the start codon (ATG) (see Fig. 5), stability of which has been shown recently to be correlated with gene expression [23].

Interestingly, the median transition temperature, given by 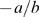, was 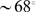
C. Although the majority of the transition temperatures lie outside the temperature range experienced by mesophiles, it is important to note that this temperature indicates when the probability of openness is 0.5. Although, the relationship between degree of openness and translation initiation is positively correlated, there exists no quantitative estimate of this relationship. The above values indicate that the RBS needs to be open only a small fraction of time for translation initiation of most genes to meet their target protein production rates.

**Figure 6:**
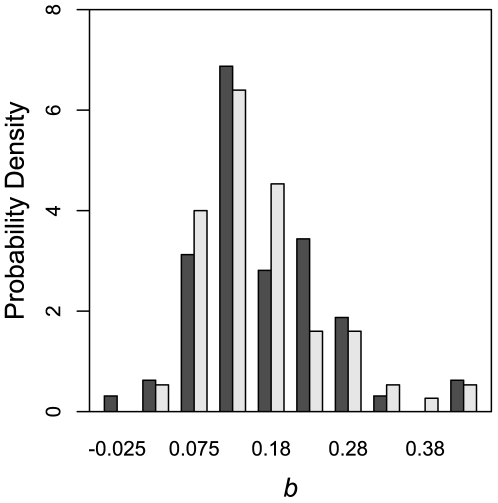
Distribution of significant 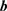 values of 76 *E. coli* genes and 64 *rpoH* genes of mesophilic 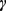-proteobacteria. The two distributions are not significantly different from each other (*Kolmogorov-Smirnov test* 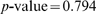).

In order to show the generality of the above results, we compared the distribution of 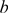 of *rpoH* of 81 mesophilic 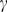-proteobacteria to that of the 100 randomly selected genes. Surprisingly, of the 81 *rpoH* sequences, 17 (21%) showed no significant change in their 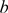. We also found that the mean of the two distributions are not significantly different from each other (*Kolmogorov-Smirnov test* 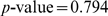), further supporting our conclusions. Fig. 6 shows the distribution of 76 *E. coli* genes with significant 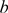 values alongside the significant 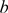 values of *rpoH* genes of 64 mesophilic 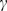-proteobacteria.

## Discussion

We present here a novel method of studying the melting of RNAs with temperature by incorporating the entire distribution of the RNA structures at a given temperature. This approach is more holistic as it takes into account the probability of finding the RNA in a sub-optimal structure based on its free energy as opposed to previous studies which have looked at structures with the least free energies only [2], [3], [8], [9], [24]. Although using the minimum free energy structure makes the analyses of structural features easier, it ignores the sub-optimal, yet highly likely structures that the RNA molecule can also adopt. Using the minimum free energy structure also becomes progressively problematic with increasing temperatures. It has been shown that as temperature increases, probability of finding the RNA in the minimum free energy structure becomes smaller [11] as at higher temperatures, various secondary structures become equally probable as the energy landscape becomes shallower and flatter. Thus, for RNAs whose structure changes with temperature, it becomes important to sample from the entire distribution of structures. In addition, since our approach is not biased towards any particular structural feature, it can be used to identify novel thermosensitive structures.

As one would expect, we find that mRNAs have an inherent tendency to melt with an increase in temperature. This tendency varies with the sequence and the difference in temperatures. Contrary to our expectations, we find that RNA thermometers are not unique with respect to their ability to increase their RBS exposure with temperature. Since it is difficult and expensive to demonstrate the effect of temperature on the RNA secondary structure in the laboratory, researchers have focused primarily on known RNA thermometers. However, due to a lack of such studies on non-thermometers, it has been hard to ascertain whether thermosensing properties are unique to a special class of RNAs. Our results call for further experimental exploration of ‘non-thermometers’ with changes in temperatures, before firm conclusions can be drawn regarding the uniqueness of RNA thermometers.

Physiological similarities between RNA thermometers and non-thermometers with respect to their melting with temperature, raise an important question that if a large number of mRNAs show an extensive increase in RBS exposure with temperature, why don't we see corresponding changes in their protein expressions. In other words, why do physiological similarities not lead to functional similarities? This discrepancy could be explained, in part, by the fact that the amount of protein expression depends on a variety of factors such as mRNA abundance and stability, amount of regulatory proteins, the stability of the protein itself, and factors apart from the accessibility of the RBS of the mRNA to the ribosome. Hence, although temperature may not result in significant phenotypic effects of certain genes in terms of protein expression, it does not preclude the possibility of changes in its RBS exposure. Thus, the above results indicate that increased RBS exposure does not solely define as to what constitutes an RNA thermometer.

One of the key challenges in such studies is to devise appropriate measures that quantify the structural features in analyzing the distribution of secondary structures. Here, we use a simple measure of openness to quantify the changes in the structure with temperature. In order to quantify complex structural features like stems and loops in a distribution of RNA structures, more sophisticated measures could be developed. Our analysis based on the current state of RNA folding algorithms is also limited by the simple energy model as well as parameter estimates used in most algorithms.

Another key limitation of this study is the fact that current RNA folding algorithms do not take into account the effect of presence of ribosome on the mRNAs secondary structure. The secondary structure of an mRNA becomes a constantly changing environment due to the presence and movement of ribosomes along the mRNA affecting the openness of a window both upstream and downstream of its current position. Hence, including the effect of ribosomes on the mRNA on translation initiation in the folding algorithm may be important in identifying RNA thermometers computationally. This is likely to be a non-trivial task both mathematically and computationally. However, we believe that incorporating the movement of ribosomes in RNA folding routine would open new avenues of research in investigating and understanding not only the effect of ribosome on the RNA structure and in translation initiation but also on the effect of any RNA-protein interactions on the secondary structure of the RNA.

## Supporting Information

Table S1 List of *Escherichia coli* genes used in the study, their functions and methods used to identify their lengths and ribosome binding sites.
(0.03 MB XLS)

Table S2 List of Mesophilic γ-proteobacteria whose *rpoH* gene sequences were used in the study.
(0.02 MB PDF)

Figure S1 The distribution of MLE estimates of b of the 75 genes that differed significantly from zero in *E. coli. rpoH*, *ibpA* and *agsA* genes show an increase in openness with temperature with b values 0.109, 0.137 and 0.0, respectively. However, none of these values are significantly higher than the mean b = 0.158, of the distribution (Wilcox test, p-value = 0.781, 0.500 and 0.958, respectively). In addition, when the base G71 is removed from *ibpA* sequence, the MLE estimate of b reduces to 0.
(0.35 MB EPS)
