# Supplementary material for: Is Thermosensing Property of RNA Thermometers Unique?
Source: PLoS One. 2010 Jul 2;5(7):e11308. doi: 10.1371/journal.pone.0011308 (PMC2896394; doi:10.1371/journal.pone.0011308)
Supplement: Table S2 — List of Mesophilic γ-proteobacteria whose rpoH gene sequences were used in the study. (0.02 MB PDF) [file pone.0011308.s002.pdf]

Aeromonas hydrophila ATCC7966
Alcanivorax borkumensis SK2
Alcanivorax borkumensis SK2
Baumannia cicadellinicola
Buchnera aphidicola (Baizongia pistaciae)
Buchnera aphidicola Sg
Buchnera sp. APS
Candidatus Blochmannia floridanus
Candidatus Blochmannia pennsylvanicus str. BPEN
Candidatus Pelagibacter ubique HTCC1062
Chromohalobacter salexigens DSM 3043
Colwellia psychrerythraea 34H
Erwinia carotovora atroseptica SCRI1043
Escherichia coli 536
Escherichia coli CFT073
Escherichia coli O157:H7 EDL933
Escherichia coli UTI89
Escherichia coli W3110
Francisella tularensis subsp. holarctica
Francisella tularensis subsp. tularensis FSC 198
Haemophilus ducreyi 35000HP
Haemophilus influenzae 86 028NP
Haemophilus influenzae KW20 Rd
Haemophilus somnus 129PT
Hahella chejuensis KCTC 2396
Hahella chejuensis KCTC 2396
Idiomarina loihiensis L2TR
Idiomarina loihiensis L2TR
Legionella pneumophila Lens
Legionella pneumophila Paris
Legionella pneumophila Philadelphia 1
Legionella pneumophila Philadelphia 1
Mannheimia succiniciproducens MBEL55E
Methylococcus capsulatus Bath
Nitrosococcus oceani ATCC 19707
Pasteurella multocida PM70
Pasteurella multocida PM70
Photobacterium profundum SS9
Photorhabdus luminescens TTO1
Pseudoalteromonas atlantica T6c
Pseudoalteromonas haloplanktis TAC125
Pseudomonas aeruginosa PAO1
Pseudomonas entomophila L48
Pseudomonas fluorescens Pf-5
Pseudomonas fluorescens PfO-1
Pseudomonas putida KT2440

Pseudomonas syringae DC3000
Pseudomonas syringae pv B728a
Pseudomonas syringae pv phaseolicola
Psychrobacter arcticum 273-4
Psychrobacter cryohalolentis K5
Saccharophagus degradans 2-40
Salmonella enterica Choleraesuis
Salmonella enterica Paratyphi ATCC9150
Salmonella enterica serovar Typhi CT18
Salmonella typhimurium LT2 SGSC1412
Shewanella denitrificans OS217
Shewanella oneidensis MR-1
Shigella boydii Sb227
Shigella dysenteriae Sd197
Shigella flexneri 2a 2457T
Shigella sonnei Ss046
Sodalis glossinidius str. morsitans
Thiomicrospira crunogena XCL-2
Vibrio cholerae El Tor N16961
Vibrio parahaemolyticus RIMD 2210633
Vibrio vulnificus CMCP6
Wigglesworthia glossinidia brevipalpis
Xanthomonas axonopodis pv. citri 306
Xanthomonas campestris 8004
Xanthomonas campestris pv. armoraciae 756C
Xanthomonas campestris pv. campestris ATCC33913
Xanthomonas campestris pv. vesicatoria str. 85-10
Xanthomonas campestris pv. vesicatoria str. 85-10
Xanthomonas oryzae KACC10331
Xanthomonas oryzae pv. oryzae MAFF 311018
Xanthomonas oryzae pv. oryzicola BLS256
Xylella fastidiosa Temecula1
Xylella fastidiosa Temecula1
Yersinia pestis biovar Medievalis 91001
Yersinia pseudotuberculosis IP32953
